# A cross-sectional regional study looking at the factors responsible for the low COVID-19 vaccination rate in Nigeria

**DOI:** 10.11604/pamj.2022.41.114.30767

**Published:** 2022-02-09

**Authors:** Onyekachi Ezekiel Ekowo, Chibuzo Manafa, Ruphina Chidiebere Isielu, Chinedu Michael Okoli, Ibe Chikodi, Azuka Favour Onwuasoanya, Sylvia Tochukwu Echendu, Ifeanyichukwu Ihedoro, Uchenna Dean Nwabueze, Obinna Chidubem Nwoke

**Affiliations:** 1 Department of Surgical Sciences University of Edinburgh, Edinburgh, Scotland,; 2 Royal Stoke University Hospitals, Stoke-on-Trent, England,; 3 Department of Public health, University of Nigeria, Enugu, Nigeria,; 4 Department of Medical Laboratory Science, Alex Ekweme Federal Teaching Hospital, Abakaliki, Ebonyi State, Nigeria,; 5 Department of Family Medicine, Federal Medical Centre, Owerri, Nigeria,; 6 Department of Ophthalmology, University of Nigeria Teaching Hospital, Ituku/Ozalla, Enugu, Nigeria,; 7 Department of Paediatrics, Nnamdi Azikiwe University Teaching Hospital, Nnewi, Anambra State, Nigeria,; 8 Department of Surgery, Federal Medical Center, Umuahia, Abia State, Nigeria,; 9 Department of Accident and Emergency, Nnamdi Azikiwe University Teaching Hospital, Nnewi, Anambra State, Nigeria,; 10 Department of Pharmacology, University of Nigeria, Enugu Campus, Enugu, Nigeria

**Keywords:** COVID-19 virus, COVID-19 vaccine hesitancy, public health

## Abstract

**Introduction:**

COVID-19 vaccination has been rolled out in Nigeria, with low uptake often attributed to shortage of the vaccine. We set out to find out the current trend so far and to the best of our knowledge, our study is one of the early studies since the roll out in the region looking at the real situation on ground. This will guide multidisciplinary decision making at increasing uptake of the vaccine.

**Methods:**

this is a descriptive cross-sectional study in the 5 South-Eastern States in Nigeria. A structured questionnaire was given to the members of the public to answer themselves or via the help of an interviewer. Data was analysed in SPSS and associations between variables compared using Chi square.

**Results:**

there are 1283 respondents in this study. Of this number, only 105 (8.2%) have had at least one of the vaccine doses. Stated reasons for not having been vaccinated are side effects (n=370, 31.5%), access to a vaccination centre (n= 239, 20.4%) and belief in one’s own immunity 186 (15.5). Having a health-related degree (p-value of 0.021), non-governmental employees (p-value of 0.003), private sector employees (p-value of 0.029) and public sector employees (p-value of 0.009) are associated with relatively higher vaccination rates.

**Conclusion:**

vaccination rate in Nigeria is still very low. Fear of side effect which is enhanced by mystical thinking is the leading factor for low turnout not just shortages. All forms of employed jobs, age and higher qualification all have significant p-values (p<0.05) and associated with higher uptake of the vaccine.

## Introduction

The low vaccination rate in Nigeria and Africa at large when compared to their richer counterparts is often attributed to vaccine shortages. The UN Secretary General has expressed his concerns regarding what he termed “vaccine distribution uneven and unfair” when speaking out on the fact that nearly 75% of the COVID-19 vaccinations worldwide has taken place in just 10 countries [1]. Even the Duke Global Health Institute did say “Will low-income countries be left behind when the COVID-19 vaccine arrives [2]. However, the real question is: is the low vaccination rate so far in Nigeria and Africa in general merely due to access to the COVID-19 vaccine? Several studies had tried to assess if people will take up the vaccine when it becomes available [3,4]. This is one of the few studies which has looked at the current trend in the ongoing vaccination so far in Nigeria. The initial roll out of the vaccine in Nigeria only invited frontline health workers and older people, however, this has been extended to all and sundry [1]. Part of this reason is due to the daily wastages in the health facilities not just in Nigeria but in most African nations [5] as vaccination turn up rate is less than what was forecasted by some fore-runner surveys which tried to see the public perception and acceptance of the COVID-19 vaccines when they become available [3,4]. There are several public health strategies to combat the pandemic including social distance, wearing face masks and adequate and effective hand washing [6]. However, vaccination is one factor which has recently proven to be a good strategy to see us out of this pandemic through its ability to reduce spread [7-9], reduce hospitalisations and severe disease [10] and significantly reduce death [11].

A study has suggested that the abundance of the vaccine is simply not enough if we truly want to maintain the targeted vaccination rate to attain herd immunity [12]. We can argue that the COVID-19 did not hit Nigeria as hard [13] as it did the UK [14] and India [15]. This does not mean we should adopt a “let´s wait and see how it goes” approach. If not for anything, we should learn from the mistakes of other nations. The REACT-1 round 12 study has proven that we are not out of the woods yet as the delta variant which was first discovered in India has replaced the UK alpha variant (Kent variant) with increased infection rates in people younger than 50 years. Thankfully, death rates are still low thanks to the protection offered by the vaccine, however, hospital admissions are gradually beginning to rise again [16]. There are two important natural (unmodifiable) factors that can be loosely associated with comparatively lower death rates and lower disease burden in this pandemic -warm weather [17] and younger age [18]. These are two factors Nigeria has in abundance no thanks to its low life expectancy of about 61 years and median age of 18.4 years [19]. However, like its natural crude deposit, it is stretching its luck far too much. The association of warmer climate to reduced spread has a very low evidence and the authors have strongly suggested that nations pursue a favourable health policy rather than “taking the easy way out” [17]. How about Long COVID-19 (having symptoms of fatigue, body aches, fever, and cough for more than 4 weeks after COVID-19 diagnosis)? About 1 million people are currently having this condition in the UK [20]. If the death rate is not that severe in Africa´s most populous nation, the sequelae of having COVID-19 alone can be debilitating. There are 3 pillars set out by the WHO in April 2020 to combating this pandemic-diagnostics, treatment, and fair vaccine distribution to all the corners of the earth. COVAX, coordinated by Gavi, is at the core to implementing the latter [21]. It set out to ensure that every nation irrespective of their resource gets a fair and equitable share of the vaccine. In a similar move, the UK has made a pledge of 100 million COVID-19 vaccines to poorer nations [22]. These are laudable. However, without strategies aimed at addressing vaccine hesitancy (refusing to be vaccinated despite the presence of vaccines) [23,24] in Nigeria, these record-breaking miles stones will not achieve its aim of vaccinating approximately 70% of the populace to gain the much needed herd immunity [12,25].

**Objectives:** 1) to determine the vaccination rate of the COVID-19 vaccine in the South-Eastern part of Nigeria and compare this to the national figure; 2) to understand the core reasons why the reported national vaccination rate remains lower than those of the developed nations..

## Methods

**Study design:** this is a descriptive cross-sectional study that was conducted between the 25^th^ of May 2021 and the 30^th^ of June 2021 in the South-Eastern region of Nigeria.

**Setting:** this study was conducted in the state capitals of the 5 South-Eastern States of Nigeria namely Enugu, Anambra, Imo, Ebonyi and Abia states.

**Target population, inclusion/exclusion criteria:** the target population was adults 18 years and above in the five South-Eastern States of Nigeria. These were generally made of people in the public who were willing and consented to be interviewed. Because no personal identifiable markers were sought, ethical approval was not needed. Only clearly filled and legible questionnaires were included. Incompletely filled or illegible questionnaires were discarded.

**Data sources:** pre-validated questionnaires were used in this study. The questionnaires were distributed in-person randomly to members of the public face-to-face. Two major methods were adopted during the questionnaire distribution process: interviewer-administered and self-administered questionnaires. The participants were given the option to choose from both options. While the non-literate participants generally chose interviewer-administered questionnaires (which means the interviewer gets to read out the questions while the responder gets to supply the relevant answers), the participants who could read and write generally chose the self-administered option.

**Study questionnaire development:** the questionnaire was designed by the research team. The final draft was tested for validity by revered academic members. A pilot test was then run by 50 participants and feedback were collected to improve the overall delivery and ease of understanding of the questionnaire by the participants. This resulted in the final questionnaire which was presented to the participants. The results from the pilot study were discarded.

**Variables:** the questionnaire contained 16 questions and were divided into four sections: section 1 involved questions about patient´s demographics (eight questions in total), section 2 assessed for Knowledge about COVID-19 (three questions), section 3 assessed the participants knowledge of COVID-19 Vaccine (two questions) and then finally, section 4 reviewed whether the participants had been vaccinated or not and the reasons for each case (three questions).

**Bias:** we do realise that interviewers are a potential source of bias. To eliminate this, we compared data from interviewers with those from patients who filled the questionnaires themselves to see if there were any differences, and there was no statistical difference in both.

**Sample size:** the sampling method was random and single staged. Only respondents who agreed to fill the questionnaires were included in the study. The minimum sample size was calculated using the link below. This gave a value of about 377 at 5% precision, 95% confidence interval and 50% response distribution [26].

**Statistical analysis:** data analysis was done using the IBM SPSS Statistical Tool version 28. The median and percentages were the mode of univariate analysis used. Chi-square test was used to assess the differences between respondents´ willingness to receive the COVID-19 vaccines. In addition, multinomial logistic regression was performed to model the relationship between a set of predictors and the subjects´ willingness to receive COVID-19 vaccines. Statistical significance was considered at P≤0.05.

## Results

**Socio-demographic characteristics of the study:** a total of 1283 respondents completed the questionnaire either by themselves (n= 960, 74%) or by the help of an interviewer (n= 323, 25.2%). The median age for this study is 26 to 45 years (n = 575, 44.8%). However, it is worth mentioning that the 65 years and above age group make up a paltry 60 respondents (4.7%). Majority of the respondents were females (n = 711, 55.4%), of Christian religion (n = 1223, 95.3%), reside in Nigeria (n = 1281, 99.8%), have a high school qualification as their highest level of academic qualification (n = 529, 41.2%). The number of respondents from the 5 South-Eastern States of Nigeria are not too varied with Enugu State being the highest with a total of 288 respondents (22.4%) and Imo state having the least with 190 respondents (14.8%).

Table 1 shows the knowledge of the virus and the vaccine amongst the respondents. Of the knowledge of the virus, nearly every single one of the respondents (n = 1271, 99.1%) have heard of COVID-19 virus. Also, 1179(91.7%) know that it is a viral illness. When asked to suggest one single way COVID-19 can be spread, the suggested answers were contact with an infected person (n = 806, 62.8%), contact with infected surfaces (n = 324, 25.3%), handshake (n = 63, 4.9%), not washing hand (n = 7, 0.5%), no social distance (n = 4, 0.3%), not wearing face mask (n = 11, 0.9%). Other answers were mosquito bites (n = 49, 3.8%), sexual contact (n = 16, 1.2%), and vaccine (n = 2, 0.2%). Of the knowledge of the COVID-19 vaccine, majority of the respondents (n = 1199, 93.5%) are aware that there is a vaccine for the COVID-19 virus, however greater number of the respondents (n = 820, 63.9%) do not know what kinds of vaccines are available in Nigeria. Only 105 (8.2%) of the respondents have been vaccinated. The reason given for being vaccinated was the same in all the respondents - protection of self and others. Of the unvaccinated respondents, the main reasons for not being vaccinated are side effects (n = 370, 31.5%), not knowing a vaccination centre (n = 239, 20.4%), waiting for an invitation to be vaccinated (n = 131, 11.2%), believe in one´s immunity being enough for protection (n = 239, 20.4%), and that it is a ploy to reduce the population of Africans (n = 117, 10%). Least reasons for not being vaccinated include believe that COVID-19 is unreal (68, 5.8%), the vaccine is a controlling memory chip (n=32, 2.7%) and being a nursing mother (n=3, 0.3%).

**Table 1:** knowledge of COVID-19 virus and the vaccine

Variables	Frequency n (%)
Have you heard of COVID-19 virus?	
Yes	1271(99.1)
**What is COVID-19?**	
Bacteria	46(3.6)
Fungus	12(0.9)
I do not know	49(3.8)
Virus	1176(91.7)
**Suggest one way the disease can be contacted**	
Not washing hands	7(0.5)
Contact with infected surface	324(25.3)
Contact with infected person	806(62.8)
Handshake	63(4.9)
Mosquito bites	49(3.8)
No social distance	4(0.3)
Not wearing face mask	11(0.9)
Sexual contact	16(1.2)
Vaccine	2(0.2)
**Have you heard of a vaccine for COVID-19?**	
Yes	1199(93.5)
**COVID 19 vaccine available in your area**	
I do not Know	820(63.9)
I know at least a vaccine type	463(36)
**Have you been Vaccinated?**	
Yes	105(8.2)
**If yes, why?**	
Protection of self and others	89(100.0)
**If no, why?**	
COVID is not real	68(5.8)
My immunity is enough	186(15.9)
Waiting for invitation	131(11.2)
I do not know a centre	239(20.4)
It is a memory chip	32(2.7)
Religious reasons	27(2.3)
Nursing mother	3(0.3)
Side effects	370(31.5)
It is a ploy to reduce our population	117(10.0)

Table 2 is a cross table looking at how the reasons why people have not been vaccinated compared with their gender and highest level of qualification. When compared to level of academic qualification, there is a difference in the pattern of the reasons for not being vaccinated. Respondents with graduate, postgraduate and health related qualifications have four major reasons for their low vaccination turn out. For graduate holders, side effect (n = 164, 29.5%), access to a vaccine centre (n = 78, 14.4%) or the vaccine itself (n = 57, 12.4%) and believe in one´s own immunity (n= 67, 14.9%) were the major reasons topping the chart. Other reasons such as COVID-19 is not real (n= 20, 4.5%), the vaccine is a memory chip (n=10, 2.2%), religious reasons (n = 9, 2.0%), nursing mother (n = 2, 0.4%), and it is a ploy to reduce our population (n = 42, 9.4%) were the least answers in the chart. This pattern of answering is similar in people with health related and post graduate qualifications. Conversely, respondents with no formal education and only a first school living certificate answered a bit differently. For no formal education, COVID-19 is not real (n = 5, 17.9%) and side effects (n = 5, 17.9%) top the chart while other reasons are distributed nearly equally. For first school leaving certificate holders, COVID is not real (n = 16, 21.9%), belief in one´s own immunity (n = 15, 20.5%) and it is a ploy to reduce our population are the top reasons in the chart. For respondents with high school qualification, we can see a pattern of answering roughly midway between respondents with graduate education and above and people with first school leaving or no formal education. Amongst these respondents, side effects (n = 150, 29.5%), access to a vaccination centre (n = 125, 24.6%), belief in one´s own immunity (n = 80, 15.7%) and it is a ploy to reduce our population (n = 52, 10.2%) are the major reasons for not being vaccinated. When the reason for not having been vaccinated is compared with gender, there is a nearly equal proportion seen in all the fields as shown in the table below.

**Table 2:** reason for not being vaccinated compared to socio-demographic characteristics

	Why have you not been vaccinated?								
Highest level of qualification	COVID is not real	Belief in one's own immunity	I know a vaccination venue but have not been invited yet	I do not know any COVID vaccination centre	It is a memory chip	Religious reasons	Nursing mother	Side effects	It is ploy to reduce our population
No formal education	5(17.9%)	4(14.3%)	2(7.1%)	4(14.3%)	2(7.1%)	2(7.1%)	0(0.0%)	5(17.9%)	4(14.3%)
First school leaving	16(21.9%)	15(20.5%)	4(5.5%)	10(13.7%)	3(4.1%)	2(2.7%)	0(0.0%)	9(12.3%)	14(19.2%)
High school	24(4.7%)	80(15.7%)	48(9.4%)	125(24.6%)	16(3.1%)	13(2.6%)	0(0.0%)	150(29.5%)	52(10.2%)
Graduate	20(4.5%)	67(14.9%)	57(12.7%)	78(17.4%)	10(2.2%)	9(2.0%)	2(0.4%)	164(36.5%)	42(9.4%)
Postgraduate	2(2.4%)	15(17.9%)	12(14.3%)	19(22.6%)	1(1.2%)	1(1.2%)	0(0.0%)	29(34.5%)	5(6.0%)
Health related degree	1(3.2%)	5(16.1%)	8(25.8%)	3(9.7%)	0(0.0%)	0(0.0%)	1(3.2%)	13(41.9%)	0(0.0%)
**Cumulative**	68(5.8%)	186(15.9%)	131(11.2%)	239(20.4%)	32(2.7%)	27(2.3%)	3(0.3%)	370(31.5%)	117(10.0%)
**Gender**									
Female	32(4.9%)	118(18.1%)	72(11.0%)	126(19.3%)	22(3.4%)	16(2.5%)	3(0.5%)	202(30.9%)	62(9.5%)
Male	36(6.9%)	68(13.1%)	59(11.3%)	113(21.7%)	10(1.9%)	11(2.1%)	0(0.0%)	168(32.3%)	55(10.6%)
**Cumulative**	68(5.8%)	186(15.9%)	131(11.2%)	239(20.4%)	32(2.7%)	27(2.3%)	3(0.3%)	370(31.5%)	117(10.0%)

On bivariate analysis, Table 3 gives a summary of the factors playing a role in the vaccination rates in the South-Eastern Nigeria. Age (p-value of 0.003), level of qualification (p<0.001) and place of work (p = 0.001) play a significant role at determining vaccination rates. One limitation of this study is that employs the services of interviewers to help ask the questions in situations where respondents are not able to do so themselves. However, mode of collection (p = 0.420) was not associated with any significance which is expected to rule out bias from the field agents who helped with collection of data. Gender, religion, place of residence, all have non-significant P-values and do not play a role at determining who gets vaccinated or not.

**Table 3:** vaccination rate compared with socio-demographic characteristics

Have you been vaccinated?		No	Yes	P-value
**Mode of collection**	Interviewer	300 (92.9)	23 (7.1)	0.420
	self	878(91.5)	82(8.5)	
**Age**				
	18 - 25	437(95.6)	20(4.4)	0.003
	26 - 45	517(89.9)	58(10.1)	
	46 - 64	171(89.5)	20(10.5)	
	65 and above	53(88.3)	7(11.7)	
**Gender**				
	Female	657(92.4)	54(7.6)	0.391
	Male	521(91.1)	51(8.9)	
**Religion**				
	Christianity	1121(91.7)	102(8.3)	0.789
	Islam	32(94.1)	2(5.9)	
	Traditional	20(95.2)	19(4.8)	
	Others	5(100.0)	0(0.0)	
**Country of residence**				
	Others	2(100.0)	0(0.0)	0.673
	Nigeria	1176(91.8)	105(8.2)	
**State of residence**				
	Abia	256(92.8)	20(7.2)	0.219
	Anambra	250(92.3)	21(7.7)	
	Ebonyi	243(94.2)	15(5.8)	
	Enugu	256(88.9)	32(11.1)	
	Imo	173(91.1)	17(8.9)	
**Highest level of qualification**				
	First school leaving	72(94.7)	4(5.3)	<0.001
	Graduate	459(91.1)	45(8.9)	
	High school	501(94.7)	28(5.3)	
	MBBS	32(72.7)	12(27.3)	
	No formal education	29(93.5)	2(6.5)	
	Postgraduate	85(85.9)	14(14.1)	
**Place of work**				
	NGO employee	32(80.0)	8(20.0)	<0.001
	Private sector employee	142(89.3)	17(10.7)	
	Public sector employee	246(85.4)	42(14.6)	
	Retired	2(100.0)	0(0.0)	
	Self employed	262(93.9)	17(6.1)	
	Student	386(95.5)	18(4.5)	
	Unemployed	108(97.3)	3(2.7)	

On multiple logistic regression analysis (Table 4), having a health-related degree (p-value of 0.021), non-governmental employees (p-value of 0.003), private sector employees (p-value of 0.029) and public sector employees (p-value of 0.009) all have significant differences and associated with relatively higher vaccination rates.

**Table 4:** multi-nominal logistic regression showing factors associated with higher vaccination rates

Have you been Vaccinated?	Yes		
	Std. error	P-value	95% C. I
**Age**			
65 and above	Ref		
18 - 25	0.529	0.134	0.783 - 6.219
26 - 45	0.447	0.364	0.625 - 3.602
46 - 64	0.483	0.577	0.508 - 3.373
**Gender**			
Male	Ref		
Female	0.215	0.519	0.753 -1.752
**Highest level of educational qualification**			
Postgraduate	Ref		
First school	0.633	0.521	0.434 - 5.185
Graduate	0.335	0.349	0.710 - 2.637
High school	0.415	0.773	0.500 - 2.542
Health related degree	0.460	0.021	0.141 - 0.852
No formal education	0.838	0.929	0.209 - 5.567
**Place of work**			
Unemployed	Ref		
NGO employee	0.728	0.003	0.029 - 0.497
Private sector employee	0.656	0.029	0.066 - 0.864
Public sector employee	0.638	0.009	0.054 - 0.662
Self employed	0.641	0.205	0.126 - 1.559
Student	0.670	0.323	0.139 - 1.919

## Discussion

Although COVID-19 is relatively a new disease and most of our learning has been from our own failures in the pandemic, some important facts are nearly certain at this point-we know that vaccine works. Vaccination reduces infection rate [7-9], reduces hospitalisation in COVID-19 related respiratory syndromes [10] and death rates from COVID-19 [11]. This study has identified a vaccination rate of 8.2% amongst the respondents (n = 105/1283, 8.2%) for people who have had at least one of the jabs. This is clearly less than the 70% target by the Nigerian federal government [1]. The official number of people who have had at least one of the COVID-19 jabs in Nigeria stands at 2.23 million as of the 23^rd^ of June 2021 according to our world in data. This represents about 0.1% of the populace [13]. This contrasts to those of the developed nations such as the UK where nearly 81.9% of the population has had one of the vaccines and 59.8% has had both in the same period [14]. About 95% of the respondents have heard of COVID-19 while about 90 percent are aware of a vaccine for COVID-19 virus. Some studies put the knowledge of COVID-19 virus at a similar figure [27,28]. The truth is that most people want to be vaccinated [3,25]. How come the vaccine uptake is not as rapid as one would expect?

In this study, concerns about side effects (n= 370, 31.5%), access to a vaccination centre (n = 239, 20.4%), belief in one´s immunity (n = 186, 15.9%) and waiting for invitation to be vaccinated (n = 131, 11.2%), are the most important factors identified. Side effects from vaccine has been a recognised driving factor to vaccine hesitancy [29-31]. This study has shown that the level of one´s educational qualification plays a significant role in their tendency to receive the COVID-19 vaccine with higher vaccination rates amongst people with MBBS (n = 12, 27%), graduate (n = 45, 8.9%) and post graduate qualifications (n = 14, 14.1%) when compared to people with only high school (n = 28, 5.3%), first school leaving (n = 4, 5.3%) or no formal education (n=2, 6.5%). This is the case in a similar study [30]. Also, people in employed jobs (NGOs, private sector, and public sector) have higher vaccination rates than people who are self-employed, unemployed or students the study has shown. This contrasts with a finding in Jordan where employed respondents were less likely to be vaccinated [32]. The older the individual, the more likely they are to accept a vaccine. This is reflected in similar studies [30-33]. How do we get out of the situation and increase the uptake in Nigeria? The internet and social media are a major source of information for people these days and often misinformation cannot be rule out [34,35]. It is suggested that the authorities should channel people to a more evidence based source of information such as television and government website [36]. Also engaging with the people through their community and church leaders will help in strengthening trust amongst the populace [37]. It is important to address the concerns of the local people rather than dispelling their concerns as merely superstitious and senseless [29]. Although COVAX and the UK have pledged their commitment in vaccinating Africa, local production of the vaccine will tackle the issue of access [38]. The AstraZeneca and Johnson & Johnson vaccines are the main vaccines in Nigeria now [1]. With the whole media campaign which makes it appear as though the AstraZeneca is an inferior brand given the lower efficacy of 62.1% compared to the Pfizer/BioNTech efficacy of 95% [39] plus the side effect profile (blood clots) of AstraZeneca vaccine, people in Nigeria may see this as a battle of them versus us as AstraZeneca is the main brand in Nigeria. COVAX should ensure that all vaccine brands are made available to every nation.

A study conducted in Denmark and Norway showed a very marginal increase in cerebral venous thrombosis (11 cases per 100,000 people) with AstraZeneca vaccines compared to the normally expected rate (2.5 cases per 100,000). It, however, did not find any excess in arterial events (heart attacks or strokes). They went further to support the EU and UK medical regulator decisions that the side effects are too low and that the benefits far exceeded the risks and countries who refused AstraZeneca vaccines would have prevented some unnecessary deaths [40]. Landmark studies such as these, quite frankly do not get media attention, even when they need to be made available to governments and ordinary people all over the world to help address the fears and concerns that people have. Early trials on the COVID-19 vaccines in Africa raised some ethical issues amongst some Africans who think Africans are only being used. However, this is not the case as only 2% of all vaccine trials take place in Africa [41]. These facts need to be made very clear to people. Also, all over Africa, there seems to be a band wagoning in the decisions of the leaders who played herd mentality to their “colonialist” counterparts. Hence, it was not about what works for Abuja but what London says. We went into a lockdown, with no reason, no strategy, and no economic cushioning as was the case in the west. And like its inception, we only relaxed things when London relaxed things [42]. This made many to lose confidence in anything the government had to say further [28]. We need consistencies in policies and strategies that reflect local need.

**Limitation of this study:** interviewers helped in the filling of the questionnaire in 323 (25.2%) respondents with the possibility of introducing bias.

## Conclusion

The low vaccination rate in Nigeria is often loosely associated with shortage even when the situation on ground suggests differently. The fear of side effects is the main reason reducing the uptake of the vaccine not just shortages. We found a vaccination rate of 8% in this study. Factors associated with higher uptake of the vaccine include: all forms employed jobs (non-governmental employees, p-value of 0.003, private sector employees, p-value of 0.029, public sector employees, p-value of 0.009), age (with higher vaccination rates in the 26-64-year-old group, p-0.003), higher qualification (p<0.001). To boost the vaccination rate, education and awareness campaign will be very useful.

### What is known about this topic

- Most Nigerians (75%) showed willingness to accept the COVID-19 vaccines before its arrival. However, government data shows a 0.1% vaccination;
- The low vaccination is attributed to poverty and vaccine shortages in Nigeria (DGHI);
- Vaccination reduces infection rate, reduces hospitalisation in COVID-19 related respiratory syndromes and death rates from COVID-19. This study has identified a vaccination rate of 8.2% amongst the respondents (n = 105/1283, 8.2%) for people who have had at least one of the jabs.

### What this study adds

- The low vaccination rate in Nigeria is mainly because people are scared about side effects fuelled by mystical thinking not necessarily due to shortages. This needs to be addressed if we truly want to vaccinate Africa in this pandemic. Individuals with higher educational qualification and people in employed jobs are the main groups who tend to be vaccinated. We can argue that these two groups of individuals have better access to objective information. This shows that education will be a useful strategy to boost vaccination.
